# Effects of Prebiotic Gum Arabic Under Antibiotic-Containing Conditions in Atopic Dermatitis-Associated Bacteria: In Vitro Evaluation and Development of Semisolid Topical Carriers

**DOI:** 10.3390/antibiotics15040378

**Published:** 2026-04-08

**Authors:** Derya Doğanay, Esra Mertoğlu, Ahmet Arif Kurt, Batuhan Cenk Özkan, Ertuğrul Osman Bursalıoğlu, Mustafa Eray Bozyel, Reyhan Aliusta, Özlem Türkoğlu, Halise Betül Gökçe, Emine Kızılay, Fatih Hacımustafaoğlu, Şaban Kalay, Rana Hamdemir, Ismail Bayır, Ismail Aslan

**Affiliations:** 1 Department of Pharmaceutical Microbiology, Faculty of Pharmacy, University of Health Sciences, Istanbul 34668, Türkiye; derya.doganay@sbu.edu.tr; 2 Department of Phytotherapy, Hamidiye Institute of Health Sciences, University of Health Sciences, Istanbul 34668, Türkiye; 241002116@ogrenci.sbu.edu.tr; 3 Polisome R&D Pharmaceutical Industry Trade Company, Isparta 32000, Türkiye; ahmetkurt@sdu.edu.tr; 4 Department of Pharmaceutical Technology, Faculty of Pharmacy, Suleyman Demirel University, Isparta 32000, Türkiye; 5 ATABIO Technologies Co., Ltd., Teknopol Istanbul, Istanbul 34903, Türkiye; batuhan.ozkan@atabio.com.tr; 6 Department of Medical Services and Techniques, Vocational School of Health Services, Sinop University, Sinop 57000, Türkiye; ebursalioglu@sinop.edu.tr; 7 SFA R&D Private Health Services Co., Ltd., Teknopark Blv, No:1 3A Z01, Teknopark Istanbul, Pendik, Istanbul 34890, Türkiye; eray.bozyel@sfaarge.com; 8 Venar Sağlık A. S., Büyükesat Mah., Çayhane St., No: 35/A GOP, Çankaya, Ankara 06700, Türkiye; reyhanaliusta@venar.com.tr; 9 Clinic of Radiology, Sancaktepe Şehit Prof. Dr. İlhan Varank Training and Research Hospital, Istanbul 34785, Türkiye; ozlem.turkoglu@sbu.edu.tr; 10 HBA Pharma Industry and Trade Inc., Sogutozu Mah., 9 Eylul Ave. No: 4/1, Cankaya, Ankara 06510, Türkiye; betul.aslan@afsu.edu.tr; 11 Department of Pharmaceutical Technology, Faculty of Pharmacy, Afyonkarahisar Health Sciences University, Afyonkarahisar 03030, Türkiye; 12 Department of Medical Services and Techniques, Hamidiye Vocational School of Health Services, University of Health Sciences, Istanbul 34668, Türkiye; emine.kizilay@sbu.edu.tr; 13 Department of Medical Services and Techniques, Hamidiye Vocational School and Techniques, University of Health Sciences, Üsküdar, Istanbul 34668, Türkiye; fatih.hacimustafaoglu@sbu.edu.tr; 14 Division of Biochemistry, Department of Basic Pharmaceutical Sciences, Faculty of Pharmacy, University of Health Sciences, Istanbul 34668, Türkiye; saban.kalay@sbu.edu.tr; 15 Pharmacology Programme, School of Cardiovascular & Metabolic Health, University of Glasgow, Glasgow G12 8QQ, UK; 2948835h@student.gla.ac.uk; 16 Department of Veterinary Medicine, Vocational School, Erzincan Binali Yıldırım University, Erzincan 24100, Türkiye; ismail.bayir@sbu.edu.tr; 17 Department of Pharmaceutical Technology, Hamidiye Faculty of Pharmacy, University of Health Sciences, Istanbul 34668, Türkiye

**Keywords:** gum arabic, acacia gum, atopic dermatitis, antibiotics, dysbiosis, *Staphylococcus aureus*, probiotics, rheology, IVRT

## Abstract

**Background/Objectives**: Atopic dermatitis (AD) is associated with gut dysbiosis linked to early-life antibiotic use and *Staphylococcus aureus* colonization. Gum Arabic (GA), a prebiotic, may modulate this dysbiosis and influence AD-related microbial balance. This study evaluated whether GA could support AD-associated probiotics-*Lactobacillus casei*, *Bifidobacterium bifidum*, and *Bifidobacterium infantis*-under amoxicillin- or azithromycin-containing conditions, examined the response of *S. aureus* under the same screening conditions, and developed GA-phospholipid-based semisolid carriers for topical application. **Methods**: Probiotic strains were cultured with 1–5% GA in the presence and absence of antibiotics, and viable cell counts were assessed. Sixteen topical formulations containing propylene glycol or isopropyl myristate in a hydrogenated phosphatidylcholine base were prepared and screened for rheological properties and galactose release using in vitro release testing (IVRT) and HPLC-UV. **Results**: GA at 1–2% concentrations promoted probiotic growth in antibiotic-free conditions. GA preserved *B. infantis* viability under azithromycin exposure in this in vitro screening model. For *S. aureus*, numerical CFU differences were observed between antibiotic-only and GA-containing conditions; however, the present screening design was not intended to determine antibiotic interaction outcomes. Formulations F14 (2% GA + 7% IPM) and F15 (3% GA + 7% IPM) exhibited optimal spreadability. IVRT showed that 6 h cumulative galactose release varied by formulation (F6 > F10 > F14 > F15). **Conclusions**: GA demonstrated dose-dependent prebiotic activity and preserved *B. infantis* viability under azithromycin exposure in this in vitro screening model. For *S. aureus*, the observed CFU differences between antibiotic-only and GA-containing conditions should be considered exploratory only and do not allow for conclusions regarding interference with antibiotic efficacy. Optimized GA-HPC systems with suitable rheological and release characteristics represent promising candidates for further preclinical investigation.

## 1. Introduction

Atopic dermatitis (AD) is a chronic inflammatory skin pathology affecting approximately 20% of the global pediatric population [1,2]. Characterized by severe pruritus, xerosis, and localized inflammation, the disease emerges from a complex interplay between genetic predisposition and environmental factors, ultimately leading to epidermal barrier disruption and systemic immune dysfunction [2,3].

Recent studies have revealed a direct correlation between AD, alongside other allergic diseases developed later in life, and gut dysbiosis (alteration in microbial composition) caused by childhood antibiotic exposure [4]. Antibiotic exposure during early life, particularly in infancy, rapidly disrupts the composition and diversity of the gut microbiota, which is already being shaped by factors such as delivery mode and diet [5]. Broad-spectrum antibiotics suppress not only pathogens but also commensal microorganisms essential for maintaining immune tolerance, thereby inducing long-term detrimental effects on mucosal immunity and epithelial integrity [6,7,8]. The resulting dysbiosis can shift the immune response toward a more allergic, Th2-dominant axis, creating a biological foundation for diseases like AD [9]. Indeed, shifts in specific intestinal taxa, such as *Clostridia*, have been linked to the onset of AD via eosinophilic inflammation [10]. Although epidemiological interpretations can be confounded by reverse causality and by indication, early antibiotic use is widely regarded as a significant trigger that, in conjunction with genetic and environmental factors, increases AD risk through the microbiota–immune development axis [11,12].

Infancy is a critical developmental window for microbiota maturation. Systemic antibiotic administration during this phase causes a dramatic reduction in the *Bifidobacterium* population—a keystone genus crucial for gut health that degrades human milk oligosaccharides and produces short-chain fatty acids (SCFAs)—leading to a dysbiotic state. In children with AD, this dysbiosis extends beyond numerical shifts, manifesting as functional impairments such as reduced fermentative capacity and pathological increases in amino acid metabolism [4]. Recent metagenomic and metabolomic studies have demonstrated that antibiotic exposure permanently alters not only the microbial composition but also its SCFA production capacity. The depletion of SCFAs, particularly butyrate and propionate, adversely affects epithelial barrier proteins like filaggrin (FLG), thereby increasing epidermal permeability and allergen penetration [13,14,15]. Immunologically, children with AD exhibit elevated levels of epithelial-derived alarmins (TSLP, IL-33) alongside a Th2-dominant cytokine profile (IL-4, IL-5, IL-13) [16]. SCFAs, primarily butyrate, modify chromatin structure via histone deacetylase (HDAC) inhibition to suppress inflammatory gene expression while simultaneously upregulating *FOXP3* expression to promote regulatory T (Treg) cell differentiation and systemic immune tolerance [17,18]. Furthermore, microbiota-derived metabolites influence microRNA (miRNA) expression, and the dysregulated miRNA profiles observed in AD have been shown to directly impact barrier proteins and cytokine production [19].

The intestinal dysbiosis in AD patients is characterized by a decrease in beneficial species (*Lactobacillus*, *Bifidobacterium*) and an increase in pathogenic load (*Escherichia coli*, *Clostridium difficile*, *Staphylococcus aureus*). Similarly, the skin microbiota is associated with an expansion of the *S. aureus* population. Increased *S. aureus* colonization contributes to the disruption of the natural microbiota and the weakening of the skin barrier through various mechanisms. This dysbiotic process activates signaling pathways via the “gut-brain-skin axis,” compromising epidermal integrity and exacerbating inflammation [2]. The influence of the gut microbiota on skin physiology is mediated not only by the immune system but also through microbial metabolites. SCFAs entering the systemic circulation can modulate keratinocyte function and the expression of epidermal barrier proteins [20,21]. Experimental models indicate that SCFAs enhance the expression of epidermal differentiation proteins like filaggrin and loricrin while suppressing NF-κB-mediated inflammatory pathways [15,21]. Consequently, the reduction in SCFA production following antibiotic use may indirectly weaken both intestinal and epidermal barriers, thereby contributing to AD pathogenesis [15].

In pediatric patients requiring mandatory antibiotic therapy, developing strategies to preserve the microbiota and restore its functional capacity is of critical importance. The enhanced SCFA production resulting from the fermentation of prebiotics like GA can reprogram host–microbiota interactions at the epigenetic level, thereby limiting immune deviations. On the other hand, *S. aureus* colonization, which frequently increases in AD and triggers immune responses in the gut, leading to disease exacerbations, poses severe clinical challenges such as biofilm formation and antibiotic resistance [1,2,22]. Given that prebiotics, when used with probiotics, can suppress pathogen adhesion and biofilm formation [23], GA may merit investigation as a microbiota-supportive adjunct under antibiotic-containing conditions; however, any effect on antibiotic efficacy, synergy, or antagonism must be established by dedicated interaction studies rather than inferred from screening-level observations.

In this context, Gum Arabic (GA), derived from *Acacia senegal* and *Acacia seyal* species, emerges as a potent prebiotic candidate capable of reversing existing dysbiosis [24]. GA is a highly branched heteropolysaccharide-protein complex exhibiting antioxidant, anti-inflammatory, anticancer, and antimicrobial properties [25,26,27]. Its biological efficacy varies depending on its botanical origin, with Sudanese varieties reportedly demonstrating stronger antimicrobial activity due to differences in secondary metabolite ratios [28,29]. In addition, GA is a heterogeneous arabinogalactan–protein biopolymer composed predominantly of branched polysaccharide fractions with a minor protein-containing fraction. Current structural models describe GA as containing arabinogalactan, arabinogalactan–protein, and glycoprotein fractions that differ mainly in molecular size and protein content, with a β-(1 → 3)-galactopyranosyl backbone and β-(1 → 6)-linked side chains terminating in arabinose, rhamnose, and glucuronic acid residues [29,30]. In the gastrointestinal tract, GA acts as a robust prebiotic, selectively fermented by colonic bacteria to stimulate *Bifidobacterium* and *Lactobacillus* populations while suppressing pathogens such as *Clostridium* [31,32]. Mechanistically, GA promotes the production of SCFAs like butyrate, which inhibits the NF-κB signaling pathway and downregulates pro-inflammatory cytokines such as IL-6 and TNF-α [27,33]. Additionally, GA has been shown to exhibit a synergistic effect with *Lactobacillus* strains, increasing antimicrobial protein stability and disrupting cell membrane integrity in multidrug-resistant (MDR) pathogens by enhancing DNA leakage [32]. This fermentation process strengthens the gut barrier and restores the Th1/Th2 immune balance, thereby modulating the gut–skin axis [1].

While the “antibiotic + supportive/adjuvant approach” appears rational in a clinical context where early-life antibiotics indirectly affect AD pathogenesis by disrupting the microbiota, synergies are not guaranteed, and certain combinations may even induce antagonism [34]. Amoxicillin and azithromycin were selected as model antibiotics because they are among the most commonly prescribed antibacterial agents in children and represent two mechanistically distinct classes, namely β-lactams and macrolides [35,36]. This pair was also considered clinically informative because azithromycin has been associated with measurable alterations in pediatric gut microbial diversity, thereby allowing for the evaluation of GA under two different antibiotic pressure profiles [36]. Therefore, quantifying the interaction window of prebiotic candidates like GA with antibiotics in vitro is crucial. Accordingly, GA should be investigated primarily as a microbiota-supportive adjunct, whereas any synergistic or antagonistic interaction with antibiotics must be confirmed by dedicated interaction assays such as checkerboard or time-kill testing [37]. Moreover, in the “microbiome-friendly” product development paradigm, the in vitro efficacy of postbiotic-containing formulations has been evaluated; specifically, their integration into liposome-gel platforms has been comprehensively characterized in terms of rheology, stability, release kinetics, and antimicrobial activity [38,39]. The systematic investigation of rheological behavior and applicability in topical liposome-gel systems aligns directly with the approach of incorporating GA into a suitable semisolid vehicle [40]. As a natural biopolymer, GA can serve as a functional platform material dictated by its rheological and kinetic parameters [26]. Furthermore, recent studies screening the antimicrobial efficacy of encapsulated or complex systems (e.g., encapsulated hemp seed oil, natural-based sunscreens) provide methodological continuity for the microbiological evaluation pipeline designed in this study [41,42].

To translate this robust microbiological and immunological potential into clinical utility, GA must be integrated into an appropriate pharmaceutical carrier system. In conditions with impaired epidermal barrier integrity like AD, it is critical that topical formulations possess a low-shear viscosity to prolong residence time at the application site while simultaneously exhibiting a pronounced shear-thinning profile to facilitate spreadability upon rubbing. Moreover, the controlled and predictable release of active prebiotic components from the semisolid matrix is the primary determinant of targeted microbiota modulation and therapeutic efficacy. Accordingly, the rheological and pharmacokinetic (in vitro release testing, IVRT) characterization of semisolid systems combining hydrogenated phosphatidylcholine (HPC) with specific penetration enhancers (PG, IPM) is essential for optimizing GA as a topical agent.

The primary objective of this study was to evaluate whether GA, as a prebiotic, could support selected AD-associated probiotic strains (*Lactobacillus casei*, *Bifidobacterium bifidum*, and *Bifidobacterium infantis*) under exposure to commonly used pediatric antibiotics (amoxicillin and azithromycin), and to examine whether GA altered the observed response of *Staphylococcus aureus* ATCC 29213 under the selected antibiotic conditions. A second objective was to translate the microbiological findings into GA-based semisolid carrier systems for topical use and to identify candidate formulations through rheological screening and in vitro release testing (IVRT) using galactose equivalents as a release marker.

## 2. Results

### 2.1. Interaction of Gum Arabic and Antibiotics on Atopic Dermatitis-Associated Microbiota

In this study, the proliferation levels of *L. casei*, *B. infantis*, *B. bifidum*, and *S. aureus* ATCC 29213 (reference pathogen strain) were compared in culture media containing varying concentrations of GA (1%, 2%, 3%, and 5% *w*/*v*) both in the presence and absence of antibiotics (azithromycin, amoxicillin). For control purposes, the baseline growth levels of these bacteria in standard media lacking both antibiotics and GA were also determined. The obtained data are presented in Table 1.

#### 2.1.1. Prebiotic Promotion and Protection of Probiotic Viability

The baseline growth level of *L. casei* in the MRS medium was 8.3 × 10^5^ CFU/mL. The addition of 1% (*w*/*v*) GA yielded a comparable count (8.4 × 10^5^ CFU/mL), whereas 2% and 3% (*w*/*v*) GA increased the count to 5.1 × 10^7^ CFU/mL, corresponding to an approximately 2-log increase. At 5% (*w*/*v*) GA, the count returned to the 10^5^ CFU/mL range. Under the selected assay conditions, no detectable growth was observed at any GA concentration in the presence of azithromycin or amoxicillin.

For *Bifidobacterium infantis*, the baseline growth of 1.9 × 10^7^ CFU/mL in antibiotic-free BHI medium increased to 6.0 × 10^7^ and 6.4 × 10^7^ CFU/mL with the addition of 1% and 2% (*w*/*v*) GA, respectively, indicating growth promotion at lower concentrations. In the presence of azithromycin, bacterial counts ranged from 5.5 × 10^6^ to 1.3 × 10^7^ CFU/mL. Notably, the counts obtained with 1% and 2% (*w*/*v*) GA (8.0 × 10^6^ and 1.3 × 10^7^ CFU/mL, respectively) were higher than in the antibiotic-only condition. No detectable growth was observed in the amoxicillin-treated groups. Evaluations could not be performed in media containing 3% and 5% (*w*/*v*) GA because the physical properties of the agar no longer allowed for reliable counting.

The population of *Bifidobacterium bifidum* in antibiotic-free BHI medium was 6.7 × 10^7^ CFU/mL. With 1% and 2% (*w*/*v*) GA supplementation, the counts increased to 8.6 × 10^8^ and 9.0 × 10^8^ CFU/mL, respectively, representing an approximately 1-log increase. Under the selected assay conditions, no detectable growth was observed in the azithromycin- or amoxicillin-containing groups.

#### 2.1.2. Interaction of GA with Antibiotic-Associated CFU Changes in *S. aureus*

The baseline growth of *Staphylococcus aureus* ATCC 29213 in antibiotic-free NA medium was 3.1 × 10^9^ CFU/mL and increased to the 5.7–5.9 × 10^9^ CFU/mL range with 1–3% (*w*/*v*) GA supplementation. In media containing azithromycin or amoxicillin alone, the counts were 4.8 × 10^9^ and 6.1 × 10^9^ CFU/mL, respectively, indicating that the selected screening concentrations did not produce complete growth suppression in this assay.

In the presence of azithromycin plus 1–3% (*w*/*v*) GA, CFU counts ranged from 4.5 to 4.8 × 10^9^ CFU/mL, with the numerically lowest value observed at 2% GA. In the amoxicillin + GA groups, counts ranged from 1.4 to 3.4 × 10^9^ CFU/mL, again with the lowest value at 2% GA. Collectively, these observations indicate that, under the present screening conditions, GA did not abolish the observed antibiotic-associated reduction in *S. aureus* CFU and may influence the magnitude of that response. Because no MIC, MBC, checkerboard, or time-kill assays were performed, these data should be interpreted as screening-level observations rather than evidence of synergy, antagonism, or resistance modulation.

### 2.2. Rheological and Structural Characterization of the Designed Semisolid Carriers

Across the complete Design of Experiments (DoE) set (F1–F16), all formulations exhibited a monotonic decrease in apparent viscosity as the shear rate increased from 1.7 to 34.0 s^−1^, signifying a distinct shear-thinning behavior over the tested range.

Within the PG block, apparent viscosity at 1.7 s^−1^ ranged from 495.0–605.0 mPa·s (SD 14.9–18.1) for 5% PG and 562.5–687.5 mPa·s (SD 16.9–20.6) for 10% PG, with a comparatively modest viscosity decay toward 34.0 s^−1^ (5% PG: 315.8–386.0 mPa·s, SD 9.5–11.6; 10% PG: 392.7–479.9 mPa·s, SD 11.8–14.4). In the IPM block, increasing the IPM concentration elevated the low-shear viscosity profile and induced a steeper shear-dependent reduction: 3% IPM yielded 585.0–715.0 mPa·s at 1.7 s^−1^ (SD 17.6–21.5) and 276.7–338.1 mPa·s at 34.0 s^−1^ (SD 8.3–10.1); whereas 7% IPM produced 810.0–990.0 mPa·s at 1.7 s^−1^ (SD 24.3–29.7) and 329.8–403.0 mPa·s at 34.0 s^−1^ (SD 9.9–12.1). In both carrier blocks, under the current formulation and processing settings, the 5% (*w*/*v*) GA level trended toward the lower end of the viscosity ranges compared to the 1–3% (*w*/*v*) GA levels.

**Figure 1:**
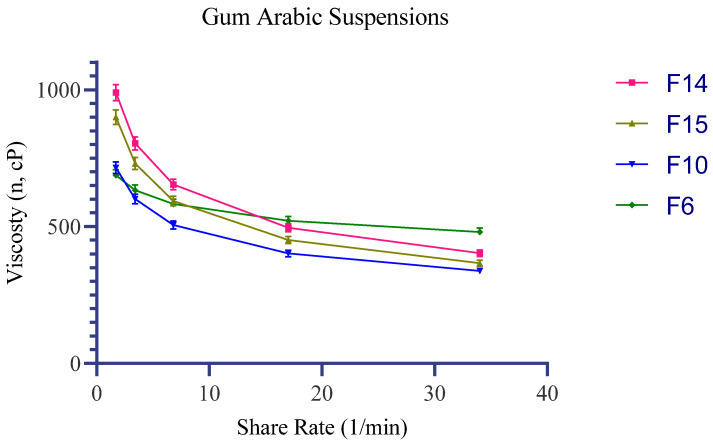
Rheogram displaying the apparent viscosity profiles of the selected formulations against shear rate (1.7–34.0 s^−1^). The data indicate shear-dependent flow behavior, with IPM-containing formulations (F14, F15) exhibiting the most pronounced shear-thinning properties.

Based on the screening objective of identifying candidates that offer higher low-shear viscosity (to support prolonged residence time at the application site) combined with a pronounced viscosity reduction at higher shear rates (to facilitate ease of spreadability), formulations F14 (2% GA + 7% IPM) and F15 (3% GA + 7% IPM) emerged as the most successful candidates. They provided the highest low-shear viscosities at 1.7 s^−1^ (990.0 mPa·s, SD 29.7 and 900.0 mPa·s, SD 27.0, respectively), alongside substantially reduced viscosities at 34.0 s^−1^ (403.0 mPa·s, SD 12.1 and 366.4 mPa·s, SD 11.0, respectively). F10 (2% GA + 3% IPM) preserved the identical qualitative shear-dependent trend but at a lower overall viscosity magnitude, serving as a well-balanced alternative within the IPM series. F6 (2% GA + 10% PG) represented the upper boundary of the PG block (1.7 s^−1^: 687.5 mPa·s, SD 20.6; 34.0 s^−1^: 479.9 mPa·s, SD 14.4) and was consequently retained as an aqueous reference for comparing PG and IPM behaviors in subsequent rheological and HPLC-based kinetic evaluations (Table 2, Figure 1).

### 2.3. In Vitro Release Kinetics and Galactose Release Profiles

IVRT showed a monotonic increase in cumulative galactose release for all formulations over the study period. At 6 h, cumulative release (mean ± SD) was 78.414% for F6, 73.186% for F10, 67.212% for F14, and 63.478% for F15. At 12 h, the highest cumulative release was observed for F6 (85.500%).

**Figure 2:**
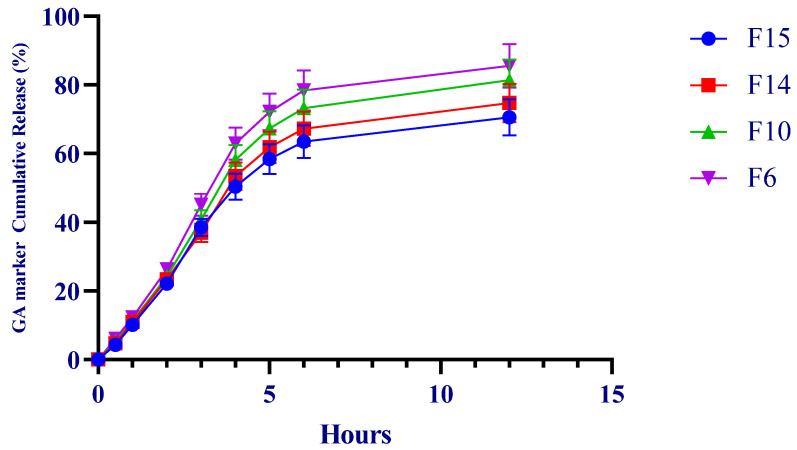
Time-dependent in vitro cumulative galactose release profiles (%) from the selected formulations in a membrane-based diffusion system over 12 h. Data are presented as mean ± SD (*n* = 3).

In kinetic screening, all formulations showed strong linearity in the Higuchi representation (cumulative release versus the square root of time), with determination coefficients (R^2^) ranging from 0.981 to 0.987. First-order transformations (% remaining versus time) also showed high linearity (R^2^ = 0.988–0.994) (Table 3, Figure 2).

IVRT is widely recognized as a discriminatory performance test for semisolid matrices, with linear release within a defined time window serving as an important suitability criterion [43,44,45]. In this context, the observed rank order suggests that PG, as a more hydrophilic cosolvent, favored faster apparent release, whereas IPM was associated with a more retarded release profile consistent with a more structured lipophilic phase.

## 3. Discussion

AD that begins early in life is often followed by food allergy, asthma, and allergic rhinitis, a progression commonly referred to as the “atopic march”. The disease reflects broader disturbances in immune regulation and skin barrier function and is increasingly prevalent in pediatric populations. Evidence from animal studies and clinical observations suggests that early antibiotic exposure may contribute to this trajectory by perturbing microbiota development during infancy, a critical period for immune and metabolic maturation [4].

One of the primary microbiota alterations observed in children subsequent to antibiotic exposure is the depletion of the *Bifidobacterium* population. *Bifidobacterium* species, which play a central role in the degradation of human milk oligosaccharides, are foundational components of the early-life gut microbiota [4]. Amoxicillin and azithromycin were selected not only because of their frequent pediatric use but also because they provide two mechanistically distinct models of antibiotic exposure, namely β-lactam and macrolide pressure [35,36]. Previous clinical evidence has shown that azithromycin can measurably alter pediatric gut microbial diversity, whereas the microbiome effect of amoxicillin appears less pronounced or less consistent in comparable settings [36]. In our study, both antibiotics exerted inhibitory effects on the tested probiotic strains under the selected assay conditions.

GA is a prebiotic dietary fiber capable of being selectively fermented by lactobacilli and bifidobacteria into short-chain fatty acids [46]. Alhssan et al. [47] demonstrated that the addition of flaxseed mucilage and GA to kefir significantly (*p* < 0.05) enhanced the growth of *Lactobacillus acidophilus* and *Bifidobacterium lactis*. Cherbut et al. [48] observed that a daily consumption of 10–15 g of GA resulted in an increase in *Bifidobacterium* and lactic acid-producing bacteria after 10 days. Similarly, Calame et al. [49] showed that a 4-week daily consumption of 10 g of GA yielded higher *Bifidobacterium* and *Lactobacillus* counts in fecal samples compared to inulin. Moreover, Ahallil et al. [31] revealed that in an in vitro colon model, GA fermentation increased *Bifidobacterium* growth while simultaneously decreasing *Clostridium* species.

*Bifidobacterium bifidum* and *Bifidobacterium infantis* were included in this study as they are natural residents of the infant gut microbiota [50]. Consistent with the literature, our findings demonstrated the prebiotic activity of GA on both species. Crucially, at the tested concentrations, GA was observed to protect *B. infantis* against the inhibitory effects of azithromycin. The absence of detectable *B. infantis* growth in the amoxicillin-containing groups in our assay is consistent with previous reports showing that bifidobacteria are generally susceptible to β-lactams, including amoxicillin, although the degree of inhibition is strain-dependent. Accordingly, the stronger inhibitory effect observed here should be interpreted as an isolate- and condition-specific finding rather than as a universal property of all *B. infantis* strains [51,52].

*Lactobacillus casei* is a probiotic strain with reported benefits in pediatric AD. Klewicka et al. [53] showed that *L. casei* DN–114001 supplementation in children with AD favorably affected gut microbiota composition, particularly bifidobacteria and clostridia. A meta-analysis by Tan-Lim et al. [54] also highlighted the potential of L. casei to alleviate allergic symptoms in pediatric AD, supporting its inclusion in the present study. Here, 2% and 3% (*w*/*v*) GA produced an approximately 2-log increase in *L. casei* under antibiotic-free conditions. However, under the selected antibiotic conditions, GA did not preserve detectable *L. casei* growth.

*Staphylococcus aureus* plays a central role in AD pathophysiology. In a study evaluating various extracts of Omani and Sudanese GA by disk diffusion, Al Alawi et al. [28] reported relatively low antibacterial activity against *S. aureus* for the n-butanol extract. In the present study, 1%, 2%, and 3% (*w*/*v*) GA alone did not reduce *S. aureus* CFU relative to the untreated condition. When combined with azithromycin or amoxicillin, numerical CFU differences were observed between antibiotic-only and GA-containing conditions, with the numerically lowest counts obtained at 2% GA in some combinations. However, because this study was not designed as a formal interaction or susceptibility assay, and because no MIC/MBC, checkerboard, FICI, or time-kill experiments were performed, these data are insufficient to determine whether GA preserves, reduces, or enhances antibiotic efficacy. Therefore, the *S. aureus* findings should be interpreted strictly as exploratory screening observations [37]. Al-Behadliy et al. [3] reported an MIC of 40 mg/mL for aqueous GA extract against *S. aureus* isolates. The absence of a direct inhibitory effect in our study may reflect differences in assay design, strain behavior, and the concentration window evaluated here. Importantly, these exploratory in vitro findings should not be used to support any clinical recommendation regarding the concomitant use of GAc with amoxicillin or azithromycin, and they should not be interpreted as evidence that GA does not interfere with antibiotic treatment in patients.

The translational phase of this study was designed to connect the microbiologically relevant GA window—particularly 1–2%—with a pharmaceutically workable semisolid carrier. In the microbiological assay, the inability to obtain reliable readings at 3–5% GA because of excessive medium softness indicated that GA affected not only biological outcomes but also matrix behavior. Accordingly, GA–HPC systems were constructed in PG and IPM blocks and screened by rheological and kinetic performance. For topical use, the target profile combines sufficient low-shear viscosity to support residence at the application site with a marked viscosity decrease under shear to facilitate spreading during rubbing. The shear-thinning behavior observed across the candidate formulations is consistent with this desired semisolid profile [55]. Within this framework, the 7% IPM systems (F14 and F15) generated the strongest low-shear structures while maintaining good spreadability at higher shear. By contrast, the PG-based series, particularly F6, showed a smaller viscosity drop across the same shear range, which is consistent with the role of PG as a hydrophilic cosolvent. In both enhancer blocks, intermediate GA concentrations (2–3%) provided the most favorable overall structural response under the present formulation conditions.

From an analytical standpoint, using galactose equivalents as a release marker instead of attempting to follow the intact GA polymer was a pragmatic approach for IVRT [56]. PMP derivatization after hydrolysis enabled sensitive quantification of liberated monosaccharides by HPLC [57]. At the same time, the robustness of this approach depends strongly on controlled hydrolysis conditions because sugar recovery and degradation can vary with hydrolysis severity [58]. The kinetic data showed a formulation-dependent release order of F6 > F10 > F14 > F15, with the faster release from F6 being consistent with the presence of 10% PG as a hydrophilic cosolvent. Previous membrane-based diffusion studies likewise indicate that PG and IPM can influence transport behavior from semisolid matrices and that acceptable fits to Higuchi-type release may be observed in selected systems [59]. Importantly, IVRT is a discriminatory in vitro tool rather than a direct surrogate for clinical performance. The strong R^2^ values obtained here indicate that the selected formulations generated reproducible, formulation-dependent release profiles within this experimental setting. These findings support the use of the selected carriers in subsequent IVPT studies with Franz diffusion cells, as well as in more mechanistic models such as co-culture and biofilm systems.

## 4. Materials and Methods

### 4.1. Microorganisms and Culture Conditions

In this in vitro study, the effects of GA on selected AD-associated probiotic strains—*Lactobacillus casei* ATA-LCC98073, *Bifidobacterium bifidum* ATA-BSP1709, and *Bifidobacterium infantis* ATA-BSI17094—were evaluated alongside *Staphylococcus aureus* ATCC 29213. The experimental setup was designed to explore whether GA could support probiotic growth under exposure to the selected pediatric antibiotics, amoxicillin and azithromycin, and whether it altered the observed response of *S. aureus* under the same conditions. All strains were preserved in 20% glycerol at −80 °C until use. Before the experiments, probiotic stock cultures were activated on De Man–Rogosa–Sharpe (MRS) agar at 37 °C for 48 h, whereas *S. aureus* was activated on Nutrient Agar (NA) at 37 °C for 24 h.

### 4.2. Antibiotics and Concentrations

Amoxicillin and azithromycin were selected as the model antibiotics because they are among the most commonly prescribed antibacterial agents in children and represent two mechanistically distinct classes, namely β-lactams and macrolides [35,36]. This pair was also considered clinically informative because azithromycin has been associated with measurable alterations in pediatric gut microbial diversity, thereby allowing for the evaluation of GA under two different antibiotic pressure profiles [36]. The specified antibiotic concentrations for the respective strains were as follows:

- For *L. casei*: amoxicillin 4 mg/L, azithromycin 1 mg/L.
- For *B. bifidum*: amoxicillin 2 mg/L, azithromycin 1 mg/L.
- For *B. infantis*: amoxicillin 2 mg/L, azithromycin 1 mg/L.
- For *S. aureus*: amoxicillin 4 mg/L, azithromycin 1 mg/L.

### 4.3. Preparation of Gum Arabic and Culture Media

Fifty grams of powdered GA (Akavital, Ankara, Türkiye) was dissolved in 100 mL of sterile distilled water to obtain a 50% (*w*/*v*) stock solution. GA is a heterogeneous arabinogalactan–protein biopolymer composed predominantly of polysaccharide fractions with a minor protein-containing fraction [29,30]. Supplier-provided batch quality information indicated compliance of the commercial grade with Eur. Ph., USP/NF, and BP specifications, including identity, physicochemical, elemental impurity, and microbiological quality criteria. The analyzed batch showed ≥90% total dietary fiber (dry basis), pH 4.80 in a 25% solution, viscosity of 70 mPa·s, moisture content of 9.1%, and total ash of 3.50% (Supplementary Material Figure S1). The material was used as supplied, and no additional lot-specific compositional profiling was performed in this study. To ensure complete extraction, the solution was held in a sealed Erlenmeyer flask for 24 h and then sterilized by filtration. Working dilutions of 1%, 2%, 3%, and 5% (*w*/*v*) were prepared from this stock [3]. The basal media were De Man–Rogosa–Sharpe agar (MRS; Condalab, Madrid, Spain) for *L. casei*, Brain Heart Infusion agar (BHI; Condalab, Madrid, Spain) for *B. bifidum* and *B. infantis*, and Nutrient Agar (NA; Condalab, Madrid, Spain) for *S. aureus*. To assess the effect of GA, each basal medium was supplemented with 1%, 2%, 3%, or 5% (*w*/*v*) GA. Media containing amoxicillin or azithromycin at the predefined strain-specific concentrations were prepared in parallel, and combined GA + antibiotic media were used to examine the corresponding screening interactions.

### 4.4. Quantitative Analysis and Spread Plate Method

Bacterial load and the effects of GA and antibiotics on the tested microorganisms were determined by the spread-plate method [60]. Fresh colonies incubated for 24 h (*S. aureus*) or 48 h (*L. casei*, *B. bifidum*, and *B. infantis*) were adjusted to 0.5 McFarland (approximately 1.5 × 10^8^ CFU/mL). One milliliter of the prepared suspension was transferred to 9 mL of 0.9% isotonic saline solution (Polifarma, Istanbul, Türkiye) to obtain a 10^−1^ dilution, and serial dilutions were continued to 10^−6^. Aliquots of 100 µL from the 10^−5^ and 10^−6^ dilutions were spread onto the relevant agar plates. Plates containing *L. casei*, *B. bifidum*, and *B. infantis* were incubated at 37 °C for 48 h under anaerobic conditions with 5% CO_2_, whereas *S. aureus* plates were incubated at 37 °C for 24 h. All experiments were performed in triplicate. After incubation, colonies were counted manually, averaged, and converted to colony-forming units per milliliter (CFU/mL) using the following formula [60]:

$$CFU/mL=\frac{Number\;of\;colonies\times Dilution\;factor}{Volume\;plated\;(\mathrm{mL})}$$

### 4.5. Translational Additional Studies: Semisolid Carrier System Development and Performance Tests

In this section, a GA-based carrier system was designed and screened in two stages to evaluate whether the biologically relevant GA window identified in the microbiology study—particularly 1–2% (*w*/*v*)—could be translated into a viable semisolid carrier. Stage 1 comprised candidate generation through preformulation and short-term physical suitability screening, and Stage 2 comprised rheological screening and IVRT-based kinetic evaluation. The semisolid performance testing strategy and IVRT window were aligned with recommended guidance for topical products [43,44,45].

#### 4.5.1. Materials

GA was sourced from Akavital (Ankara, Türkiye). Hydrogenated phosphatidylcholine (HPC; hydrogenated lecithin) was utilized to form the carrier matrix, alongside propylene glycol (PG) as a hydrophilic cosolvent and isopropyl myristate (IPM) as a lipophilic penetration enhancer. All excipients were of pharmaceutical/analytical grade; HPLC-grade solvents and reagents were used for HPLC analyses. Azithromycin and amoxicillin were selected as the model drug loads.

#### 4.5.2. GA Stock Solution and Working Concentrations

In alignment with the GA preparation approach utilized in the main study, a GA stock solution was formulated at 50% (*w*/*v*). Following a 24 h resting period in a closed vessel, it was sterilized via filtration. Working concentrations of 1%, 2%, 3%, and 5% (*w*/*v*) were subsequently prepared from this stock.

#### 4.5.3. Formulation Design (DoE) and Selection of Variables

The preformulation stage was organized as a blocked, nested screening design. The objectives were (i) to examine the impact of GA concentration on carrier microstructure and flow behavior and (ii) to compare two enhancer classes—PG as a hydrophilic cosolvent and IPM as a lipophilic enhancer—within the same phospholipid base. HPC was therefore kept constant at 2.5% (*w*/*w*), in line with reports that phosphatidylcholine/hydrogenated phosphatidylcholine can strengthen carrier architecture and support drug permeation [61,62]. The design factors were defined as follows:

- Factor A (GA, %): 1, 2, 3, 5.
- Factor B (Enhancer type; block): PG or IPM.
- Factor C (Enhancer level; nested within type): PG block at 5% (low) and 10% (high). IPM block at 3% (low) and 7% (high).
- Fixed component: HPC = 2.5% (*w*/*w*); made up to 100% with water (q.s.).

This framework generated 16 formulations (F1–F16) (Table 4). To benchmark against a robust fatty acid penetration-enhancing mechanism, an alternative optional block was defined where oleic acid (OA) was substituted for HPC at 2.5%, serving as a limited comparator set outside the main DoE; OA’s penetration-enhancing mechanism via fluidization/phase separation of stratum corneum lipids is well-documented in the literature [63,64,65].

#### 4.5.4. Preparation of Preformulations

For each formulation, the corresponding working GA solution (Factor A) was transferred to a beaker, and HPC (2.5%) was incorporated under controlled stirring until a homogeneous dispersion was obtained. PG (5% or 10%) or IPM (3% or 7%) was then added according to the design matrix. Mixing and homogenization were standardized across formulations to obtain visually homogeneous systems. The total mass was adjusted to 100% (q.s.) with sterile distilled water. The antibiotic load was kept constant across formulations to preserve comparability.

#### 4.5.5. Short-Term Physical Screening and Stability Observation

After preparation and during short-term storage (24–72 h at room temperature and 37 °C), the formulations were screened for homogeneity, phase separation/creaming, pH, and reproducibility of sampling. Systems showing rapid phase separation or inconsistent sampling behavior were not prioritized for advanced testing.

#### 4.5.6. Rheological Characterization: Apparent Viscosity-Shear Rate Profile

Flow behavior screening was performed with a Brookfield-type rotational viscometer using a multi-speed approach suitable for non-Newtonian materials [55]. A Small Sample Adapter (SC4) and an SC4-27 spindle were used, and the shear rate conversion was calculated according to the manufacturer’s adapter constant as γ˙ (s^−1^) = 0.34 × rpm. Measurements were taken at 5, 10, 20, 50, and 100 rpm, corresponding to 1.7, 3.4, 6.8, 17, and 34 s^−1^, respectively. Samples were gently homogenized before measurement to minimize air entrapment, equilibrated at 32 ± 0.5 °C for 10 min, and then subjected to the speed sweep from 5 to 100 rpm. At each speed, readings were recorded after the torque/viscosity signal had stabilized (typically 30–60 s) while keeping torque within the recommended operating range (approximately 10–100%). Measurements were performed in triplicate (*n* = 3) and reported as mean ± SD.

#### 4.5.7. In Vitro Release Test (IVRT) and HPLC Quantitative Analysis Using GA-Marker (Galactose Equivalent)

After rheological screening, formulations F6, F10, F14, and F15 were advanced to IVRT-based kinetic evaluation (*n* = 3). The experimental window was centered on the discriminatory 4–6 h interval commonly recommended for topical products; time-point sampling was therefore performed from 0 to 6 h, with 12 h included as an additional endpoint [43]. Because a Franz diffusion cell was not available, the experiments were conducted in a temperature-controlled, stirred receptor environment compatible with a membrane-based diffusion approach for semisolid performance testing [44,45]. Because GA is a complex heteropolysaccharide/glycoprotein mixture, release was monitored through a monosaccharide-marker strategy rather than by attempting to quantify an intact GA signal. As galactose is one of the dominant neutral sugar fractions obtained after GA hydrolysis, the analyte monitored in receptor samples was expressed as galactose equivalents using D-galactose standards [56].

Receptor samples were collected at 0, 0.5, 1, 2, 3, 4, 5, 6, and 12 h. To liberate monosaccharides, samples were hydrolyzed with 2 M trifluoroacetic acid (TFA) at 121 °C. Because polysaccharide hydrolysis conditions influence both sugar recovery and degradation risk, hydrolysis severity was kept constant across formulations [58]. After hydrolysis, TFA was removed, and the liberated monosaccharides were derivatized with 1-phenyl-3-methyl-5-pyrazolone (PMP) under alkaline conditions and quantified by RP-HPLC with UV/DAD detection (~245 nm). PMP-based pre-column derivatization is a well-established approach for monosaccharide analysis [57]. Cumulative percentage release was calculated for kinetic screening, and standard transformations (cumulative release versus √t, log10[% remaining] versus t, and cube root[% remaining] versus t) were generated for comparative purposes. PG and IPM have previously been reported to alter transport behavior in membrane-based diffusion experiments, and acceptable fits to the Higuchi representation may be obtained in selected systems [59].

#### 4.5.8. Statistical Approach

Given the screening-oriented design and limited replicate structure, microbiological, rheological, and IVRT data were interpreted primarily descriptively. The results are reported as mean ± SD where applicable, and formulation ranking was based on comparative evaluation of CFU counts, rheological behavior, and IVRT profiles. No formal inferential claim regarding synergy, antagonism, or susceptibility modification was made from this dataset.

## 5. Conclusions

This study provides preliminary in vitro evidence that GA, particularly within the 1–2% range, may promote the growth of selected beneficial bacteria under antibiotic-containing conditions and may support *B. infantis* viability under azithromycin exposure. For *S. aureus*, CFU differences were observed between antibiotic-only and GA-containing conditions; however, these screening-level findings are insufficient to determine whether GA preserves, reduces, or enhances antibiotic efficacy. Therefore, the present results should not be interpreted as clinical evidence and should not be used to guide patient use of GA together with these antibiotics. Dedicated MIC/MBC, checkerboard, FICI, time-kill, and subsequent in vivo/clinical studies are required before any conclusion can be made regarding antibiotic interaction.

The translational arm converted this biologically relevant concentration window into GA–HPC-based semisolid systems and screened 16 candidate formulations. Rheological profiling showed shear-thinning behavior across all candidates and identified F14 and F15 (7% IPM) as the most balanced options in terms of residence-supporting viscosity and spreadability. IVRT using galactose-equivalent monitoring differentiated the release behavior of the selected formulations in the order F6 > F10 > F14 > F15. Taken together, these data identify a rational set of candidate carriers for subsequent validation by IVPT/Franz diffusion, co-culture or biofilm models, and, ultimately, in vivo evaluation.

## Figures and Tables

**Table 1:** Comparative colony count results (CFU/mL) for bacterial proliferation in GA-supplemented media in the presence and absence of antibiotics.

*Lactobacillus casei*					
	MRS	MRS %1 GA	MRS %2 GA	MRS %3 GA	MRS %5 GA
Non-Antibiotic	8.3 × 10^5^	8.4 × 10^5^	5.1 × 10^7^	5.1 × 10^7^	5.1 × 10^5^
Azithromycin	-	-	-	-	-
Amoxicillin	-	-	-	-	-
*Bifidobacterium infantis*					
	BHI	BHI %1 GA	BHI %2 GA	BHI %3 GA	BHI %5 GA
Non-Antibiotic	1.9 × 10^7^	6.0 × 10^7^	6.4 × 10^7^	ND	ND
Azithromycin	5.5 × 10^6^	8.0 × 10^6^	1.3 × 10^7^	ND	ND
Amoxicillin	-	-	-	ND	ND
*Bifidobacterium bifidum*					
	BHI	BHI %1 GA	BHI %2 GA	BHI %3 GA	BHI %5 GA
Non-Antibiotic	6.7 × 10^7^	8.6 × 10^8^	9.0 × 10^8^	ND	ND
Azithromycin	-	-	-	ND	ND
Amoxicillin	-	-	-	ND	ND
*Staphylococcus aureus* ATCC 29213					
	NA	NA %1 GA	NA %2 GA	NA %3 GA	NA %5 GA
Non-Antibiotic	3.1 × 10^9^	5.7 × 10^9^	5.7 × 10^9^	5.9 × 10^9^	ND
Azithromycin	4.8 × 10^9^	4.8 × 10^9^	4.5 × 10^9^	4.7 × 10^9^	ND
Amoxicillin	6.1 × 10^9^	3.4 × 10^9^	1.4 × 10^9^	3.1 × 10^9^	ND

(ND: Not determined due to excessive medium softness caused by high GA concentration. (-): No growth observed).

**Table 2:** Apparent viscosity changes of the selected optimal formulations (F6, F10, F14, F15) in response to increasing shear rates (Mean ± SD, *n* = 3).

Formulation Code	Formulation Components (Single Column)	s^−1^	η (mPa·s) (3 Replicates ± SD)	Comment
F14	GA 2% + IPM 7% + HPC 2.5% + water q.s.	1.7	990.0 ± 29.7	Highest “rest” viscosity with strong shear-thinning; supports retention and good spreadability during application.
F14	GA 2% + IPM 7% + HPC 2.5% + water q.s.	3.4	804.0 ± 24.1	
F14	GA 2% + IPM 7% + HPC 2.5% + water q.s.	6.8	653.5 ± 19.6	
F14	GA 2% + IPM 7% + HPC 2.5% + water q.s.	17.0	496.0 ± 14.9	
F14	GA 2% + IPM 7% + HPC 2.5% + water q.s.	34.0	403.0 ± 12.1	
F15	GA 3% + IPM 7% + HPC 2.5% + water q.s.	1.7	900.0 ± 27.0	Similar to F14; slightly higher GA may increase film formation/adhesion while maintaining spreadability.
F15	GA 3% + IPM 7% + HPC 2.5% + water q.s.	3.4	731.0 ± 21.9	
F15	GA 3% + IPM 7% + HPC 2.5% + water q.s.	6.8	593.8 ± 17.8	
F15	GA 3% + IPM 7% + HPC 2.5% + water q.s.	17.0	451.1 ± 13.5	
F15	GA 3% + IPM 7% + HPC 2.5% + water q.s.	34.0	366.4 ± 11.0	
F10	GA 2% + IPM 3% + HPC 2.5% + water q.s.	1.7	715.0 ± 21.5	Moderate viscosity with pronounced shear-thinning; balanced candidate for a lighter sensory profile.
F10	GA 2% + IPM 3% + HPC 2.5% + water q.s.	3.4	601.2 ± 18.0	
F10	GA 2% + IPM 3% + HPC 2.5% + water q.s.	6.8	505.4 ± 15.2	
F10	GA 2% + IPM 3% + HPC 2.5% + water q.s.	17.0	401.9 ± 12.1	
F10	GA 2% + IPM 3% + HPC 2.5% + water q.s.	34.0	338.1 ± 10.1	
F6	GA 2% + PG 10% + HPC 2.5% + water q.s.	1.7	687.5 ± 20.6	Highest viscosity within the PG-series; weaker shear-thinning but good applicability in homogeneous aqueous-based systems (reference for PG vs. IPM).
F6	GA 2% + PG 10% + HPC 2.5% + water q.s.	3.4	632.7 ± 19.0	
F6	GA 2% + PG 10% + HPC 2.5% + water q.s.	6.8	582.2 ± 17.5	
F6	GA 2% + PG 10% + HPC 2.5% + water q.s.	17.0	521.5 ± 15.6	
F6	GA 2% + PG 10% + HPC 2.5% + water q.s.	34.0	479.9 ± 14.4	

**Table 3:** IVRT endpoints and Higuchi rate parameters (*n* = 3).

Formulation	Q6h (%; Mean ± SD)	Q12h (%; Mean ± SD)	kH (%·h^−1^/^2^)	R^2^ (0.5–6 h)
F6	78.414 ± 2.352	85.500 ± 2.565	44.872	0.981–0.987
F10	73.186 ± 2.196	81.350 ± 2.440	42.017	0.981–0.987
F14	67.212 ± 2.016	74.709 ± 2.241	38.330	0.981–0.987
F15	63.478 ± 1.904	70.559 ± 2.117	36.433	0.981–0.987

**Table 4:** DoE formulation matrix (HPC fixed at 2.5%; water q.s. to 100%; percentages are *w*/*w*).

Code	Block	GA (%)	PG (%)	IPM (%)	HPC (%)
F1	PG	1	5	0	2.5
F2	PG	2	5	0	2.5
F3	PG	3	5	0	2.5
F4	PG	5	5	0	2.5
F5	PG	1	10	0	2.5
F6	PG	2	10	0	2.5
F7	PG	3	10	0	2.5
F8	PG	5	10	0	2.5
F9	IPM	1	0	3	2.5
F10	IPM	2	0	3	2.5
F11	IPM	3	0	3	2.5
F12	IPM	5	0	3	2.5
F13	IPM	1	0	7	2.5
F14	IPM	2	0	7	2.5
F15	IPM	3	0	7	2.5
F16	IPM	5	0	7	2.5

## Data Availability

The raw data supporting the conclusions of this article will be made available by the authors on request.

## Supplementary Materials

The following supporting information can be downloaded at: https://www.mdpi.com/article/10.3390/antibiotics15040378/s1. Figure S1: Certificate of Analysis for Gum Arabic supplied by Akavital.

## Abbreviations

The following abbreviations are used in this manuscript:

AD	Atopic Dermatitis
BHI	Brain Heart Infusion
CFU	Colony-Forming Unit
DoE	Design of Experiments
FDA	U.S. Food and Drug Administration
FLG	Filaggrin
GA	Gum Arabic (Acacia gum)
HDAC	Histone Deacetylase
HPC	Hydrogenated Phosphatidylcholine
HPLC	High-Performance Liquid Chromatography
IPM	Isopropyl Myristate
IVRT	In Vitro Release Testing
MDR	Multidrug-Resistant
MIC	Minimum Inhibitory Concentration
MRS	De Man-Rogosa-Sharpe
NA	Nutrient Agar
NF-κB	Nuclear Factor kappa-light-chain-enhancer of activated B cells
OA	Oleic Acid
PG	Propylene Glycol
PMP	1-phenyl-3-methyl-5-pyrazolone
SCFA	Short-Chain Fatty Acid

## References

[1] Tang H., Li W., Xu Y., Zhou Y., Hamblin M.R., Wen X. (2025). Gut microbiota modulation: A key determinant of atopic dermatitis susceptibility in children. Front. Microbiol..

[2] Wrześniewska M., Wołoszczak J., Świrkosz G., Szyller H., Gomułka K. (2024). The role of the microbiota in the pathogenesis and treatment of atopic dermatitis—A literature review. Int. J. Mol. Sci..

[3] Al-Behadliy N.K., Al-Wazni W.S., Alwan A.H. (2020). Evaluation of some biological activities of Arabic gum (*Sengalia senegal*) aqueous extract in vivo and in-vitro. AIP Conf. Proc..

[4] Hoskinson C., Medeleanu M.V., Reyna M.E., Dai D.L., Chowdhury B., Moraes T.J., Mandhane P.J., Simons E., Kozyrskyj A.L., Azad M.B. (2024). Antibiotics taken within the first year of life are linked to infant gut microbiome disruption and elevated atopic dermatitis risk. J. Allergy Clin. Immunol..

[5] Azad M.B., Konya T., Maughan H., Guttman D.S., Field C.J., Chari R.S., Sears M.R., Becker A.B., Scott J.A., Kozyrskyj A.L. (2013). Gut microbiota of healthy Canadian infants: Profiles by mode of delivery and infant diet at 4 months. CMAJ.

[6] Jernberg C., Lofmark S., Edlund C., Jansson J.K. (2010). Long-term impacts of antibiotic exposure on the human intestinal microbiota. Microbiology.

[7] Round J.L., Mazmanian S.K. (2009). The gut microbiota shapes intestinal immune responses during health and disease. Nat. Rev. Immunol..

[8] Thaiss C.A., Zmora N., Levy M., Elinav E. (2016). The microbiome and innate immunity. Nature.

[9] Russell S.L., Gold M.J., Hartmann M., Willing B.P., Thorson L., Wlodarska M., Gill N., Blanchet M.-R., Mohn W.W., McNagny K.M. (2012). Early life antibiotic-driven changes in microbiota enhance susceptibility to allergic asthma. EMBO Rep..

[10] Lee E., Lee S.Y., Kang M.J., Kim K., Won S., Kim B.J., Choi K.Y., Kim B.S., Cho H.J., Kim Y. (2016). Clostridia in the gut and onset of atopic dermatitis via eosinophilic inflammation. Ann. Allergy Asthma Immunol..

[11] Metsälä J., Lundqvist A., Virta L.J., Kaila M., Gissler M., Virtanen S.M. (2013). Mother’s and offspring’s use of antibiotics and infant allergy to cow’s milk. Epidemiology.

[12] Penders J., Kummeling I., Thijs C. (2011). Infant antibiotic use and wheeze and asthma risk: A systematic review and meta-analysis. Eur. Respir. J..

[13] Brown S.J., McLean W.H.I. (2023). One remarkable molecule: Filaggrin. J. Investig. Dermatol..

[14] Langan S.M., Irvine A.D., Weidinger S. (2024). Atopic dermatitis. Lancet.

[15] Paller A.S., Kong H.H., Seed P., Naik S., Scharschmidt T.C., Gallo R.L., Luger T., Irvine A.D. (2023). The microbiome in patients with atopic dermatitis. J. Allergy Clin. Immunol..

[16] Weidinger S., Novak N. (2016). Atopic dermatitis. Lancet.

[17] Furusawa Y., Obata Y., Fukuda S., Endo T.A., Nakato G., Takahashi D., Nakanishi Y., Uetake C., Kato K., Kato T. (2013). Commensal microbe-derived butyrate induces the differentiation of colonic regulatory T cells. Nature.

[18] Arpaia N., Campbell C., Fan X., Dikiy S., van der Veeken J., deRoos P., Liu H., Cross J.R., Pfeffer K., Coffer P.J. (2013). Metabolites produced by commensal bacteria promote peripheral regulatory T-cell generation. Nature.

[19] Pessôa R., Clissa P.B., Sanabani S.S. (2023). The Interaction between the Host Genome, Epigenome, and the Gut–Skin Axis Microbiome in Atopic Dermatitis. Int. J. Mol. Sci..

[20] Trompette A., Gollwitzer E.S., Yadava K., Sichelstiel A.K., Sprenger N., Ngom-Bru C., Blanchard C., Junt T., Nicod L.P., Harris N.L. (2014). Gut microbiota metabolism of dietary fiber influences allergic airway disease and hematopoiesis. Nat. Med..

[21] Salem I., Ramser A., Isham N., Ghannoum M.A. (2018). The gut microbiome as a major regulator of the gut-skin axis. Front. Microbiol..

[22] Di Domenico E.G., Cavallo I., Bordignon V., Prignano G., Sperduti I., Gurtner A., Trento E., Toma L., Pimpinelli F., Capitanio B. (2018). Inflammatory cytokines and biofilm production sustain *Staphylococcus aureus* outgrowth and persistence: A pivotal interplay in the pathogenesis of atopic dermatitis. Sci. Rep..

[23] De Marco S., Piccioni M., Muradyan D., Zadra C., Pagiotti R., Pietrella D. (2017). Antibiofilm and Antiadhesive Activities of Different Synbiotics. J. Prob. Health.

[24] Elnour A.A.M., Nour A.H., Ishag K.E.A. (2025). Biological Applications of Secondary Metabolites Extract (SME) from Acacia Gums (AGs). Gum Arabic and Breast Cancer Biology.

[25] Adhikari D., Rangra N.K. (2023). Antimicrobial activities of the Acacia genus: A review. Asian Pac. J. Trop. Biomed..

[26] Cebeci E., Yüksel B., Aliusta R., Yılmaz Ş., Bursalıoğlu E.O., Bozyel M.E., Gökçe H.B., Kalay Ş., Kurtuluş Ş.Ö., Kurt A.A. (2026). Gum Arabic modulates redox–ionic microenvironments via rheology and kinetics to induce selective cytotoxicity in colorectal cancer cells. Gels.

[27] Safia K., Amina B., Houda T., Hamdi I. (2024). The biological activities and phytochemical investigations of acacia Senegal’s aqueous extracts of gum Arabic. Braz. Appl. Sci. Rev..

[28] Al Alawi S.M., Hossain M.A., Abusham A.A. (2018). Antimicrobial and cytotoxic comparative study of different extracts of Omani and Sudanese Gum acacia. Beni-Suef Univ. J. Basic Appl. Sci..

[29] Mahendran T., Williams P.A., Phillips G.O., Al-Assaf S., Baldwin T.C. (2008). New insights into the structural characteristics of the arabinogalactan-protein (AGP) fraction of gum Arabic. J. Agric. Food Chem..

[30] Mohamed S.A., Elsherbini A.M., Alrefaey H.R., Adelrahman K., Moustafa A., Egodawaththa N.M., Crawford K.E., Nesnas N., Sabra S.A. (2025). Gum Arabic: A commodity with versatile formulations and applications. Nanomaterials.

[31] Ahallil H., Maskat M.Y., Abdullah A., Sarbini S.R. (2020). The effect of Acacia senegal as a potential prebiotic on obese gut microbiota. Food Res..

[32] Bhola J., Bhadekar R. (2025). Enhancing antimicrobial efficacy: Gum acacia-enriched *Lactobacillus consortium* against multidrug-resistant pathogens. Med. Microecol..

[33] Mohammad S., Karim M.R., Iqbal S., Lee J.H., Mathiyalagan R., Kim Y.J., Yang D.U., Yang D.C. (2024). Atopic dermatitis: Pathophysiology, microbiota, and metabolome—A comprehensive review. Microbiol. Res..

[34] Avşar İ.S., Doğanay D., Mertoğlu E. (2025). Investigation of the combinatorial effects of metformin and selected antibiotics on *Klebsiella pneumoniae*. J. Immunol. Clin. Microbiol..

[35] Fleming-Dutra K.E., Demirjian A., Bartoces M., Roberts R.M., Taylor T.H., Hicks L.A. (2018). Variations in antibiotic and azithromycin prescribing for children by geography and specialty—United States, 2013. Pediatr. Infect. Dis. J..

[36] Oldenburg C.E., Sié A., Coulibaly B., Ouermi L., Dah C., Tapsoba C., Bärnighausen T., Ray K.J., Zhong L., Cummings S. (2018). Effect of commonly used pediatric antibiotics on gut microbial diversity in preschool children in Burkina Faso: A randomized clinical trial. Open Forum Infect. Dis..

[37] Odds F.C. (2003). Synergy, antagonism, and what the chequerboard puts between them. J. Antimicrob. Chemother..

[38] Aslan İ., Tarhan Çelebi L. (2023). Postbiotics cosmetic formulation: In vitro efficacy studies on a microbiome friendly antiperspirant. J. Res. Pharm..

[39] Gökçe H.B., Aslan İ. (2024). Novel liposome–gel formulations containing a next generation postbiotic: Characterization, rheological stability, release kinetics, and in vitro antimicrobial activity studies. Gels.

[40] Aslan İ., Aytekin A.F. (2023). Production and characterization of newly developed alcohol-free topical liposome-gel transdermal drug delivery systems containing estradiol (E2)/estriol (E3) for post-menopausal women. J. Res. Pharm..

[41] Özdemir M.N., Bursalıoğlu E.O., Doğanay D., Kurt A.A., Aslan İ. (2025). Antimicrobial activity studies of encapsulated hemp seed oil. J. Immunol. Clin. Microbiol..

[42] Kalay Ş., Güler G., Şallı E., Bozyel M.E. (2025). Development, characterization and antimicrobial potential of a new natural based sunscreen formulation. J. Immunol. Clin. Microbiol..

[43] U.S. Food and Drug Administration (2022). In Vitro Release Test Studies for Topical Drug Products Submitted in ANDAs: Guidance for Industry (Draft Guidance).

[44] United States Pharmacopeia (2023). <1724> Semisolid Drug Products—Performance Tests.

[45] Kanfer I., Rath S., Purazi P., Mudyahoto N.A. (2017). In vitro release testing of semi-solid dosage forms. Dissolution Technol..

[46] Leylak C., Özdemir K.S., Gurakan G.C., Ogel Z.B. (2021). Optimisation of spray drying parameters for *Lactobacillus acidophilus* encapsulation in whey and gum Arabic: Its application in yoghurt. Int. Dairy J..

[47] Alhssan E., Ercan S.Ş., Bozkurt H. (2023). Effect of flaxseed mucilage and gum Arabic on probiotic survival and quality of kefir during cold storage. Foods.

[48] Cherbut C., Michel C., Raison V., Kravtchenko T., Severine M. (2003). Acacia gum is a bifidogenic dietary fibre with high digestive tolerance in healthy humans. Microb. Ecol. Health Dis..

[49] Calame W., Weseler A.R., Viebke C., Flynn C., Siemensma A.D. (2008). Gum arabic establishes prebiotic functionality in healthy human volunteers in a dose-dependent manner. Br. J. Nutr..

[50] Sasaki Y., Horigome A., Odamaki T., Xiao J.Z., Ishiwata A., Ito Y., Kitahara K., Fujita K. (2021). Novel 3-O-α-D-galactosyl-α-L-arabinofuranosidase for the assimilation of gum arabic arabinogalactan protein in *Bifidobacterium longum* subsp. longum. Appl. Environ. Microbiol..

[51] Moubareck C., Gavini F., Vaugien L., Butel M.-J., Doucet-Populaire F. (2005). Antimicrobial susceptibility of bifidobacteria. J. Antimicrob. Chemother..

[52] Mancabelli L., Mancino W., Lugli G.A., Argentini C., Longhi G., Milani C., Viappiani A., Anzalone R., Bernasconi S., van Sinderen D. (2021). Amoxicillin-clavulanic acid resistance in the genus *Bifidobacterium*. Appl. Environ. Microbiol..

[53] Klewicka E., Cukrowska B., Libudzisz Z., Slizewska K., Motyl I. (2011). Changes in gut microbiota in children with atopic dermatitis administered the bacteria *Lactobacillus casei* DN–114001. Pol. J. Microbiol..

[54] Tan-Lim C.S.C., Esteban-Ipac N.A.R., Mantaring J.B.V., Chan Shih Yen E., Recto M.S.T., Sison O.T., Alejandria M.M. (2021). Comparative effectiveness of probiotic strains for the treatment of pediatric atopic dermatitis: A systematic review and network meta-analysis. Pediatr. Allergy Immunol..

[55] (2020). Standard Test Methods for Rheological Properties of Non-Newtonian Materials by Rotational (Brookfield-Type) Viscometer.

[56] Zueva O.S., Klimovitskaya M.A., Skvortsova P.V., Khair T., Kazantseva D.A., Abakumova Y., Gromova N.R. (2025). Supramolecular structure and complexation of gum Arabic in aqueous solutions: What determines its protective functions in nature and technologies?. Macromol.

[57] Vojvodić Cebin A., Komes D., Ralet M.-C. (2022). Development and validation of HPLC-DAD method with pre-column PMP derivatization for monomeric profile analysis of polysaccharides from agro-industrial wastes. Polymers.

[58] Mankarios A.T., Jones C.F.G., Jarvis M.C., Threlfall D.R., Friend J. (1979). Hydrolysis of plant polysaccharides and GLC analysis of their constituent neutral sugars. Phytochemistry.

[59] Arellano A., Santoyo S., Ygartua P. (1999). Influence of propylene glycol and isopropyl myristate on the in vitro percutaneous penetration of diclofenac sodium from carbopol gels. Eur. J. Pharm. Sci..

[60] (2023). Performance Standards for Antimicrobial Susceptibility Testing.

[61] Fujii M., Shiozawa K., Watanabe Y., Matsumoto M. (2001). Effect of phosphatidylcholine on skin permeation of indomethacin from gel prepared with liquid paraffin and hydrogenated phospholipid. Int. J. Pharm..

[62] Manca M.L., Cencetti C., Matricardi P., Castangia I., Zaru M., Diez-Sales O., Nacher A., Valenti D., Maccioni A.M., Fadda A.M. (2016). Glycerosomes: Use of hydrogenated soy phosphatidylcholine mixture and its effect on vesicle features and diclofenac skin penetration. Int. J. Pharm..

[63] Naik A., Pechtold L.A.R.M., Potts R.O., Guy R.H. (1995). Mechanism of oleic acid-induced skin penetration enhancement in vivo in humans. J. Control. Release.

[64] Rowat A.C., Kitson N., Thewalt J.L. (2006). Interactions of oleic acid and model stratum corneum membranes as seen by 2H NMR. Int. J. Pharm..

[65] Haque T., Talukder M.M.U. (2018). Chemical enhancer: A simplistic way to modulate barrier function of the stratum corneum. Adv. Pharm. Bull..

