# Supplementary material for: Effects of Prebiotic Gum Arabic Under Antibiotic-Containing Conditions in Atopic Dermatitis-Associated Bacteria: In Vitro Evaluation and Development of Semisolid Topical Carriers
Source: Antibiotics (Basel). 2026 Apr 8;15(4):378. doi: 10.3390/antibiotics15040378 (PMC13113804; doi:10.3390/antibiotics15040378)
Supplement: Supplementary file 1 [file antibiotics-15-00378-s001.zip › antibiotics-4214318-supplementary.pdf]

Figure S1. Certificate of Analysis for Gum Arabic supplied by Akavital

129 chemin de Croisset
CS 94151 - 76723 ROUEN CEDEX
FRANCE
Tél. +33 (0)2 32 83 18 18
Fax +33 (0)2 32 83 19 19

nexira
Innovation Inspired by Nature

Name of product **FIBREGUM B**

Lot number **250688**

Date of manufacture **05/09/25** **Expiry date** **05/09/28**

Place of manufacture NEXIRA - Route de Neufchâtel 76440 SERQUEUX - FRANCE

CERTIFICATE OF ANALYSIS

Test	Method	Specifications	Result
Description		White to yellowish-white powder	White to yellowish-white powder
Total dietary fibers (on dry weight) (%)	AOAC 985.29	≥ 90%	≥ 90%
pH of 25% solution	Eur.Ph	4.1 to 5.0	4,80
Color of 25% solution	Lovibond	≤ 10	8,4
Viscosity of 25% solution (mPa.s)	Brookfield LVF 60 rpm	60 to 100 mPa.s	70
Moisture (5H-105°C) (%)	USP 921 Method III	≤ 10%	9.1
Acid insoluble matters (%)	Eur.Ph	≤ 0.10%	0,04
Total ashes (%)	Eur.Ph	≤ 4.0%	3,50
Acid insoluble ashes (%)	USP 561	≤ 0.5%	≤ 0.5%
Mesh size through 63 µm (%)	Vibro sieving	≤ 15%	11,7
Solubility and reaction*	Eur.Ph	Passes test	Passes test
Glucose and fructose*	Eur.Ph	Passes test	Passes test
Starch, dextrin and agar*	Eur.Ph	Passes test	Passes test
Sterculia gum*	Eur.Ph	Passes test	Passes test
Tragacantha*	Eur.Ph	Passes test	Passes test
Tannins*	Eur.Ph	Passes test	Passes test
Identification*	Eur.Ph	Passes test	Passes test
Lead* (ppm)	ICP-OES/ICP-MS	≤ 0.1 ppm	≤ 0.1 ppm
Arsenic* (ppm)	ICP-OES/ICP-MS	≤ 0.5 ppm	≤ 0.5 ppm
Mercury* (ppm)	SAA	≤ 0.1 ppm	≤ 0.1 ppm
Cadmium* (ppm)	ICP-OES/ICP-MS	≤ 0.1 ppm	≤ 0.1 ppm
Total heavy metal* (ppm)	FCC Method II	≤ 5 ppm	≤ 5 ppm
Total plate count (CFU per g)	NF EN ISO 4833-1	≤500/g	≤500/g
Yeast and molds (CFU per g)	ISO 6611	≤100/g	≤100/g
E.coli	NF ISO 7251	ABS/5g	ABS/5g
Salmonella	NF EN ISO 6579-1	ABS/25g	ABS/25g

/**/ periodicity of the analysis as defined on the Technical Data Sheet**

Comply with the EC regulation, 231/2012
Conform to Eur.Ph, USP/NF, BP

SERQUEUX, September 12, 2025

Visa Labo Contrôle NEXIRA

The information here above is based on our current knowledge. NEXIRA cannot be hold responsible besides the guarantees written on its supply contracts, based on the fact that it does not control the final use of this product. It is the buyer's responsibility to comply with local texts and laws regulating its activity and the use of this product.

SAS au capital de 11 386 336 € - RCS ROUEN 344 770 870
